# Dynamic soft tissue mobilization versus proprioceptive neuromuscular facilitation in reducing hamstring muscle tightness in patients with knee osteoarthritis: a randomized control trial

**DOI:** 10.1186/s12891-023-06571-y

**Published:** 2023-06-02

**Authors:** Khadija Nafees, Aftab Ahmed Mirza Baig, Syed Shahzad Ali, Farhan Ishaque

**Affiliations:** 1 Department of physiotherapy, Institute of Physical Medicine and Rehabilitation, Dow University of Health Sciences, Karachi, Pakistan; 2 Department of physiotherapy, Sindh Institute of Physical Medicine and Rehabilitation, Karachi, Pakistan

**Keywords:** Manual therapies, Anterior knee Pain Syndrome, Physiotherapy, Soft tissue, Therapy

## Abstract

**Background:**

Knee osteoarthritis (KOA) considered as one of the most common degenerative diseases of synovial joint. KOA is mostly managed by physical therapy, focused on pain management, the range of motion and muscle strengthening but muscle flexibility is usually neglected. A study was conducted to evaluate the effectiveness of dynamic soft tissue mobilization (DSTM) in comparison with the proprioceptive neuromuscular facilitation (PNF) stretching in the management of hamstring tightness, reduction of pain intensity and improvement of physical functionality in KOA.

**Methods:**

Forty eight patients with KOA were randomly allocated to group A receiving DTSM and group B receiving PNF stretching. The cryotherapy and isometric strengthening exercises were also given to both groups. Total treatment duration consisted of 4 weeks, 3 sessions per week and total 12 sessions per patient. Each treatment session comprised of 30 min. At baseline and post treatment, Active knee extension test(AKET), Visual analogue scale (VAS), and Knee Injury and Osteoarthritis Outcome Score (KOOS) were used to assess hamstring flexibility, pain intensity level and physical functional capability respectively. The continuous variables were shown as mean and standard deviations. For the comparison of outcome within and between groups, paired sample and independent t-test was applied. Considerable p value was less than 0.05.

**Results:**

The between group analysis of VAS, right AKE test, and left AKE test showed non-significant (p > 0.05) mean difference as 0.2 (95% CI= -0.29, 0.70), 1.79 (95% CI= -1.84, 4.59), 1.78 (95% CI= -1.6, 5.19) respectively. KOOS domains of symptom, pain, ADLs, sports and recreational, and quality of life had also non-significant (p > 0.05) mean difference as 1.12 (95% CI= -4.05, 6.3), -5.12 (95% CI= -12.71, 2.46), -2.55 (95% CI= -7.47, 2.38), -2.7 (95% CI= -9.72, 4.3), and − 0.68 (95% CI= -7.69, 6.36) respectively. Significant (p < 0.001) improvement was shown in both groups for all outcome measures after 12 sessions.

**Conclusion:**

DSTM and PNF stretching, both treatments are equally beneficial in KOA for hamstring flexibility, pain reduction and functional mobility in terms of AKET, VAS, and KOOS respectively.

**Trial Registration:**

ClincalTrials.Gov with ID: NCT04925895, 14/06/2021, retrospectively registered.

## Background

Osteoarthritis (OA) is considered a chronic degenerative articular disorder [1]. It is a major contributory factor in pain and physical disability, especially in the elderly population. In the developed world, the literature suggests it causes chronic disability due to knee osteoarthritis (KOA) [2].

According to the Global Burden of Disease 2010, KOA is the 11th highest disease contributing to disability. It prevailed at 3.8% in almost 300 studied health conditions [3]. The burden of KOA is increasing in the southeast Asian population as in other developing parts of the globe. Preliminary data from southeast Asian countries shows that 31% of men and 35% of women had KOA with radiographic evedence [4]. Women are more prevalent to it, particularly after menopausal age [5]. Former studies show that urban population areas are more prevalent than rural ones. A locally conducted study shows that the prevalence of KOA is more in North Pakistan than in Southern Pakistan [6, 7].

Pain reduction, improvement in joint ROM, and strengthening adjoining knee muscles are primarily focused areas in Physical therapy management of KOA, [8, 9] but muscular and ligamentous tightness is ignored during treatment. A study found that knee osteoarthritis significantly affects the flexibility of the hamstring muscle [10]. These impairments affect limb function and biomechanics of the gait [11]. The application of treatment to resolve this impairment are essential and should remain key interest to research. Among different stretching techniques proprioceptive neuromuscular facilitation (PNF) stretching has a safe and efficient effect on improving hamstring flexibility. PNF stretching works on hamstring flexibility leading to pain reduction and improved mobility. PNF stretching produces an isometric contraction in the hamstring muscle. The autogenic inhibition phenomenon leads to hamstring muscle relaxation and reduced resistance during the stretch. This procedure helps to improve ROM and muscle flexibility [12]. However, another option to treat is soft Dynamic soft tissue mobilization (DSTM). It is widely believed amongst physiotherapists that soft tissue mobilization is effective option to treat decreased muscle flexibility [13]. Dynamic soft tissue mobilization (DSTM) is a soft tissue technique used to increase muscle length. It is effective on the specific tight area of muscle by combining the classic massage technique and the dynamic component of the technique [13]. It is reported that the DSTM increases hamstring flexibility [14]. As there is practical and theoretical difference that the PNF works with autogenic inhibition of the tight muscle with isometric contraction, [12] however DTSM works with reciprocal inhibition with eccentric contraction [13]. This two treatment should be identified that which treatment has better effects to treat KOA. The difference in theory and application of interventions suggests the difference in the improvement. DSTM was more effective than PNF stretching in reducing hamstring tightness and pain. Though it was a study on low back pain, they target hamstring tightness through DSTM [15]. According to authors’ knowledge there is limited evidence regarding the effects of specific treatment intervention in comparison to another. So, there is a need for an effective relaxation technique that could serve to improve hamstring flexibility issues commonly experienced by patients with KOA. The objective of this study is to evaluate the effectiveness of DSTM compared with the PNF stretching in the management of hamstring tightness, reduction of pain intensity, and improvement of physical functionality in KOA. The study hypothesized that there is a statistically significant difference between dynamic soft tissue mobilization and PNF stretching in reducing hamstring tightness in knee osteoarthritis.

## Methods

### Study design and participants

**Fig. 1:**
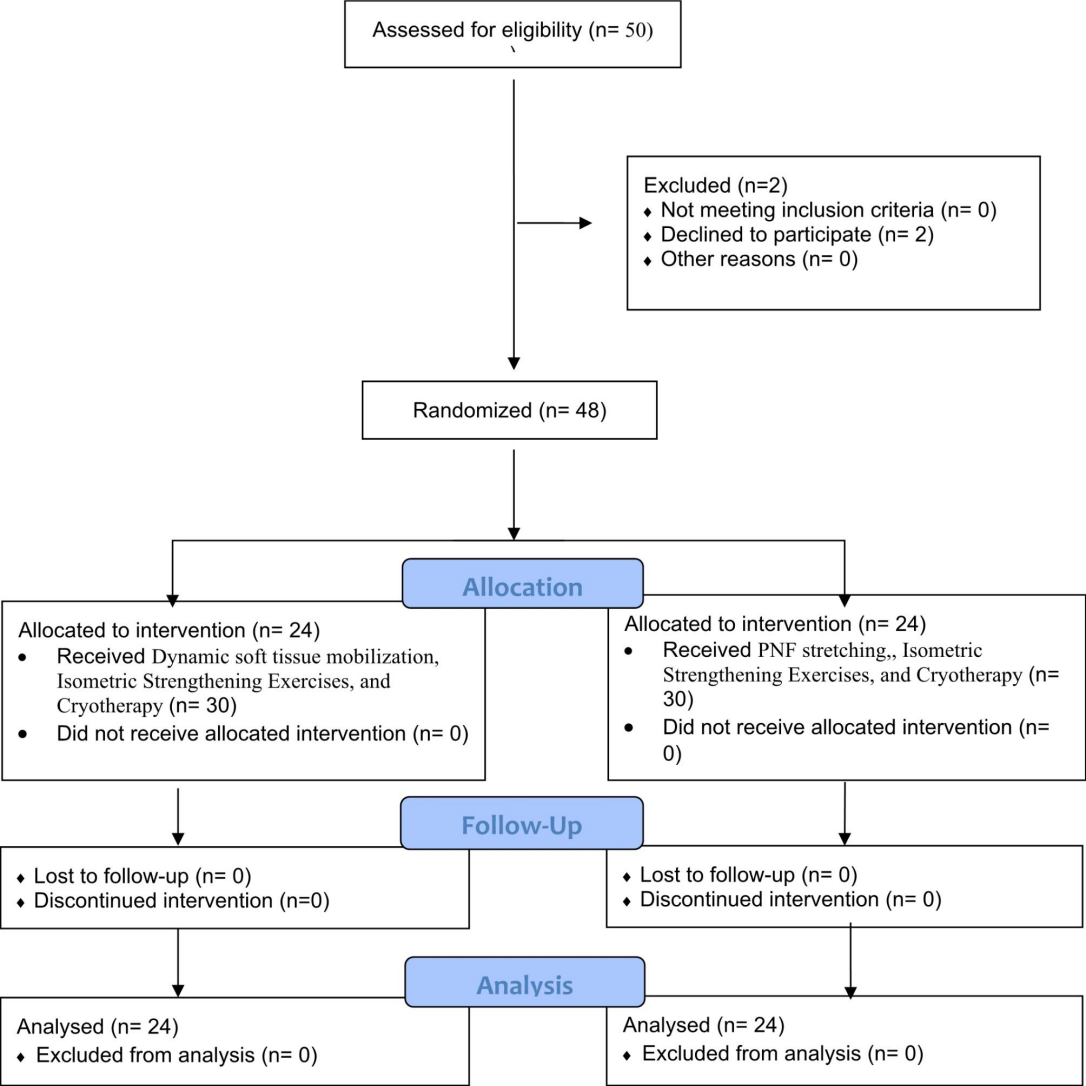
CONSORT flowchart of the recruitment, randomization and follow up of participant

This study was a single-blinded, two-arm, parallel-design, randomized control trial. It was conducted at Sindh Institute of Physical Medicine & Rehabilitation, Karachi, Pakistan, duration of April 2021 and September 2021. This study included participants 40 years and above, subjects with tight hamstring muscles, and American College of Rheumatology clinical and radiological classification criteria for KOA. However, the participants with positive SLR (sciatic nerve test), a neurological disorder that impacts the lower extremity, Musculoskeletal knee deformity e.g., varus, lower limb internal fixation, previous history of lower limb arthroplasty or any type of knee surgery, Previous history of malignancy or any infectious disease which effecting the lower extremity, any assistive device(stick/cane), Previous history of spinal surgery, and subjects having sciatica or low back pain were excluded from the study. The CONSORT flowchart is presented in Fig. 1.

### Sample size estimation

This study included a sample size of forty-eight patients (24 in each group). This was computed using software named open epi version 3, Paired Means Power Analysis with a confidence interval of 99% and 95% power of the test, mean ± S.D of VAS 5.27 ± 0.8 within group A and 3.81 ± 1.4 within group B [16].

### Randomization and envelope concealment

All participants were randomly allocated into two treatment groups with a ratio of 1:1 by an independent researcher who was unaware of the recruitment and treatment of participants (Fig. 1). The statistician generated a randomization list through software (www.random.org) for the randomization. The allocation of treatment was concealed using sealed envelopes. .

### Masking

This randomized controlled trial was assessor-blinded. The outcomes assessor in this study was blinded to the allocated treatment to the participant.

### Outcome measures

At baseline and post-treatment, the active knee extension test (AKET), Visual analog scale (VAS), and Knee injury and osteoarthritis outcome score (KOOS) were used as outcome measures for the assessment of hamstring flexibility, pain intensity level, and physical functional capability respectively. The VAS and AKET were primary outcome measures while the KOOS was a secondary outcome measure.

### Active knee extension test (AKET)

It is used in assessing the length of the hamstring muscle by using goniometry with the position of 90° of the hip flexed. For measuring hamstring flexibility, it is used as a gold standard based on the body of knowledge at present available [17]. For measuring the hamstring flexibility by AKET, the patient was positioned in a supine lying with 90˚ of flexed hip and knee. The test was conducted on the right extremity and then on the left extremity. The lateral condyle of the femur was marked for the placement of the fulcrum of the universal goniometer. The greater trochanter of the femur was kept as the reference point for the placement of the stationary arm of goniometry along with the long axis of the femur and the moveable arm was kept in line with the lateral side of the fibula pointing towards the lateral malleolus. Then the patient was asked to extend the knee joint until a mild stretch in the back of the thigh. At this point the degree of popliteal angle was recorded with average of 3 consecutive readings as the final reading for AKET outcome measurement [18]. The cut-off values for hamstring tightness consideration are different for both genders as for males, the active knee extension angle should be > 33.0˚ and for females, be > 23.4˚ [19].

### Visual analogue scale (VAS)

It is a subjective scale that is bidirectional and used for pain measurement. It consists of a 10 cm line labeling on both sides parallels. It starts with a line indicating the least “no pain” and ends with a line that indicating most “worst pain”. Every patient rated their pain on VAS. The distance between the “no pain” anchor and the patient’s mark was measured on the 10-cm line through a ruler to record VAS scores. A higher score indicates greater pain intensity. It is considered the most reliable and stable pain measurement scale. It has the least measurement error and minimal detectable change values for the pain of KOA [20].

### Knee injury and osteoarthritis outcome score (KOOS)

It is a subjective outcome measure. It contains 42 items in five subscales to check different categories i.e. pain (9 items), symptoms (7 items), functions in the activity of daily living (ADLs) (17 items), sports and recreational activities (5 items), and quality of life(4 items)0.21 All the patients were assessed with the KOOS. All subscales were scored separately through a Likert scale which had 5 options to answer from 0 (no problems) to 4 (extreme problems). The total score was considered as a percentage of the sum of each subscale’s score. It was from 0 to 100, with 0 representing extreme problems in the knee and 100 representing no problems. As per evidence, it has strong content validity, test-retest reliability, internal consistency, construct validity, and age and condition-related subscales response [22]. This study used KOOS Urdu version, which is proved with its validity and reliability among the local Pakistani population suffering from KOA [23].

**Table 1:** Baseline Characteristics of Studied Samples

Variables		Group A: DSTM (n = 24)	Group B: PNF Stretching (n = 24)	P value
Gender ^b^	Male	3 (12.5)	8 (33.3)	0.86^†^
	Female	21 (87.5)	16 (66.7)	
Age (Years) ^a^		52.08 ± 7.08	56.83 ± 8.77	0.045*
Weight (Kg) ^a^		75.38 ± 22.06	68.79 ± 9.03	0.18
Chronicity of knee OA (months) ^a^		31.54 ± 38.3	18.21 ± 23.1	0.15

DSTM: Dynamic soft tissue mobilization, PNF: proprioceptive neuromuscular facilitation

^a^Values are mean and standard deviation, ^b^ Values are frequency and percentage

^*^level of significance with Chi-square was used for association, ^†^level of significance with Independent t test

### Intervention

The interventional group ‘A’ received dynamic soft tissue mobilization technique (DSTM), and the interventional group ‘B’ received PNF stretching while cryotherapy and isometric strengthening exercises were given to both groups [24]. The trained physiotherapist with more than ten years of clinical experience in musculoskeletal conditions provided this intervention to the patients. Physiotherapist provided treatment session individually in a cubical on the treatment couch. All the actions were within ethical constraints in taking and recording outcome data. In case of any symptom aggravation, patients were allowed to take the same analgesics and muscle relaxants prescribed by the referring consultant.

Group A received twelve sessions of the DSTM with three sessions per week for four weeks. Each session took 30 min [14]. It was performed to affect the knee pain with effects on hamstring tightness.

1. Assessing the tight muscle group: The patient was prone lying. The physiotherapist applied a few deep strokes longitudinally on the hamstring muscle to assess the exact tightened part of the hamstring muscle and to limit the treatment to that specific area [14].
2. Dynamic intervention: The patient was in a supine lying with 90˚ flexion of the knee and hip joint. The physiotherapist applied the deep longitudinal strokes from the distal towards the proximal direction along with the tightened area of the hamstring muscle group while passively stretching the leg with the other hand in the direction of the hamstring lengthened position. Physiotherapist appkied five longitudinal strokes and 20 s shaking at the end of this technique [14].
3. Then progressed to the next dynamic technique in the above-mentioned sequence but physiotherapist asked the patient to do leg extension actively to achieve reciprocal inhibition of the hamstring muscle [14].
4. Then in the next technique, the patient applied a force to the physiotherapist’s hand to engage the hamstring eccentrically as the muscle was lengthened to its end range. In that position, the physiotherapist was applying 5 deep longitudinal strokes from distal towards the proximal direction on the tight hamstring muscle [14].

The use of the progression in the DTSM was concerning the treatment response of the participant.

**Table 2:** Comparison of VAS scores and AKE angle test within groups

Variables	At baseline ^a^	Post treatment ^a^	Mean difference	*D*	P value*
VAS(0–10 cm)					
DSTM group	7.52 ± 1.56	2.41 ± 0.91	5.11	14.19	< 0.01
PNF Stretching group	7.52 ± 1.47	2.21 ± 0.79	5.31	15.61	< 0.01
Right AKE test (degree)					
DSTM group	25.75 ± 8.0	7.71 ± 4.8	18.04	9.49	< 0.01
PNF Stretching group	29.5 ± 11.67	9.08 ± 6.16	20.42	7.59	< 0.01
Left AKE test (degree)					
DSTM group	27.13 ± 9.11	7.34 ± 5.06	19.79	9.33	< 0.01
PNF Stretching group	27.58 ± 10.85	9.12 ± 6.55	18.46	7.15	< 0.01

DSTM: Dynamic soft tissue mobilization, PNF : proprioceptive neuromuscular facilitation, AKE: active knee extension, VAS: visual analogue scale

^a^Values are mean and standard deviation, *level of significance using paired sample t-test

In group B, patients received the proprioceptive neuromuscular facilitation (PNF) technique of relaxation, the hold relax on tight hamstring muscle.

The position of the patient was supine lying with 90° flexion of the hip. The physiotherapist passively extended the knee joint where the patient was feeling the mild stretch. Then physiotherapist asked the patient to do knee flexion in counter-resistance applied by the therapist by using about 50% of his maximum strength and isometric contraction of the hamstring muscles was achieved. Patient maintained that isometric contraction for 8 s then relaxed on the physiotherapist’s command. Just after relaxation therapist further stretches the hamstring muscle to the point of mild to moderate painless stretch and the patient hold it for 30 s. Three repetitions were applied in each session [25].

### Interventions in both groups

Cryo therapy: Both groups received cold pack on anterior knee joint for 10 min [26].

Isometric Quadriceps Strengthening Exercises: These were given to both groups as the gold standard. The patient was lying supine. Physiotherapist placed a knee roll beneath the knee and instructed the patient to press the roll by using the back of the knee joint. The press was hold for 10 s following relaxed. Two sets of 10 repetitions were performed [27, 28].

Isometric Hip Adductor Strengthening Exercises: These were also given to both groups. The patient was lying supine with both knees flexed. Physiotherapist placed a knee roll between the knee and instructed the patient to press the roll by joining both knees. The press was hold for 10 s following relaxed. About 2 sets of 10 repetitions were performed [27, 28].

### Harms and adverse events

There are no harms and adverse event reported during the period of trial.

### Data analysis procedure

The IBM-SPSS, version 23.0 was used to store and analyzed the data. The Shapiro-Wilk test analyzed the normality. The P-value was > 0.05, so the null hypothesis is accepted showing evidence that the data is normally distributed. Mean with standard deviation were reported for baseline quantitative data sets like age (years), weight (kg), and Chronicity of KOA (months). Counts with percentages were given for gender and other qualitative data sets, and mean with standard deviation were also given for studied parameters; VAS, Active knee Extension Test (right and left), KOOS outcomes (symptoms, pain, activities of daily living, sports and recreational and quality of life) scores in both treatment groups DSTM and PNF. The chi-square test was used for the association of qualitative data. Paired sample t-test compared these parameters within the group and an independent sample t-test compared the post-treatment outcomes between groups. P-values less than 0.05 were considered statistically significant.

**Table 3:** Comparison of KOOS within groups

Variables	At baseline ^a^	Post treatment ^a^	Mean difference	*D*	P value*
KOOS Symptoms					
DSTM group	48.25 ± 23.24	84.50 ± 9.22	-36.25	-7.10	< 0.01
PNF Stretching group	51.04 ± 19.35	83.38 ± 8.61	-32.34	-7.48	< 0.01
KOOS Pain					
DSTM group	37.54 ± 14.30	73.13 ± 17.11	-35.59	-7.82	< 0.01
PNF Stretching group	45.08 ± 12.19	78.25 ± 6.93	-33.17	-11.59	< 0.01
KOOS ADLs					
DSTM group	44.96 ± 14.92	77.58 ± 9.52	-32.62	-9.03	< 0.01
PNF Stretching group	46.67 ± 16.37	80.13 ± 7.28	-33.46	-9.16	< 0.01
KOOS Sports and recreational					
DSTM group	18.96 ± 9.99	50.63 ± 12.0	-31.67	-9.95	< 0.01
PNF Stretching group	17.50 ± 12.15	53.33 ± 12.12	-35.83	-10.23	< 0.01
KOOS Quality of life					
DSTM group	21.42 ± 9.63	53.71 ± 11.21	-32.29	-10.72	< 0.01
PNF Stretching group	22.04 ± 11.12	54.38 ± 12.93	-32.34	-9.18	< 0.01

DSTM: Dynamic soft tissue mobilization, PNF : proprioceptive neuromuscular facilitation, KOOS: Knee Injury and Osteoarthritis Outcome Score, ADLs: activity of daily livings

^a^Values are mean and standard deviation, *level of significance using paired sample t-test

## Results

The comparison of the baseline characteristics between groups is tabulated. Among all variables, only age showed significant difference (p < 0.05) between groups (Table 1).

The within group analysis of both groups for mean VAS and mean Right and left Knee AKE angle showed significant (p < 0.001) improvement after 12 sessions with very large effect size (Table 2). The KOOS symptom, pain, ADLs, sports and recreational, and quality of life were also significantly (p < 0.001) improved after 12 session treatment within both groups (Table 3).

However the between group analysis showed non-significant difference (p > 0.05) for mean VAS and AKE angle test after 12 session treatment (Table 4) The KOOS symptom, pain, ADLs, sports and recreational, and quality of life had also non-significant (p > 0.05) difference after 12 session treatment between both groups (Table 5). The null hypothesis fell within the 95% confidence interval of mean difference of all variables (Tables 4 and 5).

## Discussion

This research study was conducted to determine the effects of the DSTM and PNF stretching in improving hamstring flexibility with the isometric strengthening of knee extensors and hip adductors using VAS for assessing pain intensity, AKE angle test for evaluating hamstring flexibility, and KOOS for assessing functional mobility. By comparing both groups the findings of this study showed equal effectiveness of DSTM and PNF stretching.

**Table 4:** Comparison of VAS scores and AKE angle test between groups at post treatment

Variables	DSTM group ^a^	PNF Stretching group ^a^	Mean difference (95% CI)	P value*
VAS(0–10 cm)	2.41 ± 0.91	2.21 ± 0.79	0.2 (-0.29, 0.70)	0.415
Right AKE test (degree)	172.66 ± 4.8	170.87 ± 6.16	1.79 (-1.84, 4.59)	0.393
Left AKE test (degree)	172.66 ± 5.06	170.88 ± 6.55	1.78 (-1.6, 5.19)	0.294

DSTM: Dynamic soft tissue mobilization, PNF : proprioceptive neuromuscular facilitation, AKE: active knee extension, VAS: visual analogue scale

^a^Values are mean and standard deviation, *level of significance using independent t-test

**Table 5:** Comparison of KOOS between groups at post treatment

Variables	DSTM group ^a^	PNF Stretching group ^a^	Mean difference (95% CI)	P value*
KOOS Symptoms	84.50 ± 9.22	83.38 ± 8.61	1.12 (-4.05, 6.3)	0.664
KOOS Pain	73.13 ± 17.11	78.25 ± 6.93	-5.12 (-12.71, 2.46)	0.18
KOOS ADLs	77.58 ± 9.52	80.13 ± 7.28	-2.55 (-7.47, 2.38)	0.3
KOOS Sports and recreational	50.63 ± 12.0	53.33 ± 12.12	-2.7 (-9.72, 4.3)	0.44
KOOS Quality of life	53.71 ± 11.21	54.38 ± 12.93	-0.68 (-7.69, 6.36)	0.84

DSTM: Dynamic soft tissue mobilization, PNF: proprioceptive neuromuscular facilitation, KOOS: Knee Injury and Osteoarthritis Outcome Score, ADLs: activity of daily livings

^a^Values are mean and standard deviation, *level of significance using independent t-test

In this study, the DSTM technique showed within-group improvement in pain intensity on the VAS scale with a marked reduction from baseline to end of the session, and it was greater than the minimal clinically relevant difference on VAS [29]. This improvement in the current study suggests the reciprocal inhibition effects on the relaxation of hamstring muscles leading to increase blood circulation and decreased pain. It follows the relaxation of hamstring muscles (eccentric activity) with isotonic contraction of the quadriceps. Another study found similar results of DSTM on tight hamstring muscles and significantly reduce pain by using a different outcome measure, the numerical pain rating scale but that study enrolled patients with low back pain [30]. According to the authors’ knowledge no single study is available that explored the effects of DSTM in KOA with hamstring tightness.

The PNF stretching group also showed significant improvement in pain reduction after 12 sessions of treatment, with a significant reduction of pain intensity on the VAS scale while the effect size was very large with the mean change value crossing a minimal clinically relevant difference of 0.84 cm on VAS [31]. Theoretically, this decrease in pain sensitivity might be due to decrease muscle spasms due to auto-inhibition following after isometric contraction of the tight hamstring muscle. The previous study conducted on stretching exercises in patients with KOA had less significant results in terms of pain reduction as compared to this study, [32] most probably due to the dropout ratio because of muscle soreness during treatment. Furthermore, this difference in results might be due to the difference in treatment application.

The between-group analysis of the VAS score in this study showed that there was a non-significant difference in post-treatment values i.e., PNF stretching was more effective than DSTM in pain reduction but non-significantly statistically with falling of null hypothesis within the range of 95% CI. Contradictory to this study, the results of another research showed the superiority of DSTM in reducing pain on VAS as compared to PNF stretching [30].

In this study, group A, who received DSTM, showed a significant increase in hamstring flexibility on the left and right both sides based on the AKE angle test score which is a gold standard outcome measure with intra-test reliability of 0.94 [17]. These results are well supported by another study done on a 45 participant’s sample and found that DSTM is more effective in increasing hamstring flexibility as compared to classic soft tissue mobilization group. The findings suggest that detecting the specific area of tightness in the hamstring muscle and focusing the treatment on that area through using dynamic techniques could give a significant result in achieving hamstring results [14].

The studies suggested that PNF stretching uses proprioceptive stimulation for the relaxation (inhibition) of the muscle groups by inhibiting the reflexive component in muscle contraction, increasing muscle relaxation, and later increasing joint range [29]. The findings of this study were completed in this context as AKET scores for both knees were found to be improved by PNF stretching with the level of significance.

The DSTM has a little more effectiveness on AKET score as compared to PNF stretching but in between group analysis, the average difference of both groups is not significant with the null hypothesis falling within the range of 95% CI. This proved that both treatments on average are equally effective in hamstring tightness using AKET. The previous study done on the comparison of DSTM and PNF stretching in hamstring tightness showed different results from this study as it reported that DSTM was more beneficial and had significant effects on reducing hamstring tightness in comparison with PNF stretching [15]. This difference in the results may be due to the change in population as the above study have a low back pain population and we have knee osteoarthritis patients.

As the physical activity limitation is more common in KOA so for assessing physical functions, KOOS was used as an outcome measure to take the subjective response from patients. It has 5 subscales, and it is considered an extension of WOMAC [21]. Regarding the within groups analysis, the DSTM group and PNF stretching group both showed significant improvement in all five subscales in post-treatment assessment. In the between-group analysis except for the KOOS-symptoms subscale, in all 4 subscales including KOOS-pain, KOOS-ADLs, KOOS-sports & recreational, and KOOS-QOL, the PNF stretching group showed slightly better improvement than the DSTM group after the 4 weeks treatment but non-significantly. While in DSTM, KOOS-symptoms scores are more improved than the PNF group but with a non-significant difference between the groups with falling of null hypothesis in the range of 95% CI. The fact that both groups were added with isometric strengthening exercises of quadriceps, and hip adductors should also keep in consideration that it could add more beneficence in both treatment groups. As the previous study showed significant effects of isometric strengthening exercises on muscle strength, pain reduction, and functional ability in patients with knee osteoarthritis [33]. Based on the study results we are unable to reject the null hypothesis and it is confirmed that in both techniques no one is superior to the other.

The highlighted areas of this study were the selection of the least considerable issue (hamstring tightness) in knee osteoarthritis and its treatment strategies. Some studies were found on the comparison of DSTM and PNF stretching in hamstring tightness but no one specifically on knee osteoarthritis. This study provides results for bilateral knee osteoarthritis however the previous studies had not considered bilateral knee joint osteoarthritis. In this research, the above-mentioned lacking was considered as the beneficial basis for future research. This study provides a new vision in consideration to treat hamstring tightness by DSTM and PNF stretching and can be used to maximize the treatment effect in knee osteoarthritis patients with tight hamstrings. The findings could be used to treat patients with knee osteoarthritis to decrease the osteoarthritis burden. This study also provides a base for further research ahead.

This study was limited to patients in a single clinical setting. The previous literature on the knee OA population linked to this study treatment was limited. Only primary OA patients were considered for this study. There were more females in the study sample due to some societal barriers. There was a small sample size (n = 48). Sampling was done on the non-probability sampling technique which may cause sample biases. Same-ethnicity patients were recruited which may limit the study’s generalization to the KOA population, globally.

## Conclusion

This study concluded that both approaches are significantly and equally effective in knee OA treatment. There is no one of dynamic soft tissue mobilization and PNF stretching is more beneficial than to another in reduction of hamstring tightness, decreasing pain intensity, and functional mobility improvement in knee osteoarthritis.

## Data Availability

The data is available from corresponding author on reasonable request.

## List of Abbreviations

OA: Osteoarthritis
KOA: Knee Osteoarthritis
DSTM: Dynamic Soft Tissue Mobilization
PNF: Proprioceptive Neuromuscular Facilitation
VAS: Visual analogue scale
AKE: Active Knee Extension,
AKET: Active Knee Extension Test,
KOOS: Knee Injury and Osteoarthritis Outcome Score
ADLs: Activity of daily livings
ROM: Range of Motion
